# Trends in Diagnosis Related Groups for Inpatient Admissions and Associated Changes in Payment From 2012 to 2016

**DOI:** 10.1001/jamanetworkopen.2020.28470

**Published:** 2020-12-07

**Authors:** Ty J. Gluckman, Kateri J. Spinelli, Mansen Wang, Amir Yazdani, Gary Grunkemeier, Steven M. Bradley, Jason H. Wasfy, Abhinav Goyal, Andrew Oseran, Karen E. Joynt Maddox

**Affiliations:** 1 Center for Cardiovascular Analytics, Research and Data Science, Providence Heart Institute, Providence St. Joseph Health, Portland, Oregon; 2 Healthcare Delivery Innovation Center, Minneapolis Heart Institute and Minneapolis Heart Institute Foundation, Minneapolis, Minnesota; 3 Associate Editor, *JAMA Network Open*; 4 Cardiology Division, Department of Medicine, Massachusetts General Hospital and Harvard Medical School, Boston; 5 Division of Cardiology, Department of Medicine, Emory University School of Medicine, Atlanta, Georgia; 6 Cardiovascular Division, Department of Medicine, Washington University School of Medicine, St. Louis, Missouri

## Abstract

**Importance:**

Hospitals are reimbursed based on Diagnosis Related Groups (DRGs), which are defined, in part, by patients having 1 or more complications or comorbidities within a given DRG family. Hospitals have made substantial investment in efforts to document these complications and comorbidities.

**Objective:**

To examine temporal trends in DRGs with a major complication or comorbidity, compare these findings with 2 alternative measures of disease severity, and estimate associated changes in payment.

**Design, Setting, and Participants:**

This retrospective cohort study used data from the all-payer National Inpatient Sample for admissions assigned to 1 of the top 20 reimbursed DRG families at US acute care hospitals from January 1, 2012, to December 31, 2016. Data were analyzed from July 10, 2018, to May 29, 2019.

**Exposures:**

Quarter year of hospitalization.

**Main Outcomes and Measures:**

The primary outcome was the proportion of DRGs with a major complication or comorbidity. Secondary outcomes were comorbidity scores, risk-adjusted mortality rates, and estimated payment. Changes in assigned DRGs, comorbidity scores, and risk-adjusted mortality rates were analyzed by linear regression. Payment changes were estimated for each DRG by calculating the Centers for Medicare & Medicaid Services weighted payment using 2012 and 2016 case mix and hospitalization counts.

**Results:**

Between 2012 and 2016, there were 62 167 976 hospitalizations for the 20 highest-reimbursed DRG families; the sample was 32.9% male and 66.8% White, with a median age of 57 years (interquartile range, 31-73 years). Within 15 of these DRG families (75%), the proportion of DRGs with a major complication or comorbidity increased significantly over time. Over the same period, comorbidity scores were largely stable, with a decrease in 6 DRG families (30%), no change in 10 (50%), and an increase in 4 (20%). Among 19 DRG families with a calculable mortality rate, the risk-adjusted mortality rate significantly decreased in 8 (42%), did not change in 9 (47%), and increased in 2 (11%). The observed DRG shifts were associated with at least $1.2 billion in increased payment.

**Conclusions and Relevance:**

In this cohort study, between 2012 and 2016, the proportion of admissions assigned to a DRG with major complication or comorbidity increased for 15 of the top 20 reimbursed DRG families. This change was not accompanied by commensurate increases in disease severity but was associated with increased payment.

## Key Points

**Question:**

Have the proportion of inpatient admissions assigned to a Diagnosis Related Group (DRG) with major complication or comorbidity and the associated payments changed over time?

**Findings:**

In this cohort study of US hospitalizations from 2012 to 2016, the proportion of DRGs with major complication or comorbidity increased for 15 of the top 20 reimbursed DRG families; however, commensurate increases in comorbidity burden or risk-adjusted mortality were not observed. These DRG shifts were associated with at least $1.2 billion in increased payment.

**Meaning:**

In this study, trends in assigned DRGs did not reflect trends in the underlying case mix.

## Teaser

This cohort study evaluates trends in Diagnosis Related Groups with a major complication or comorbidity and estimates associated changes in payment.

## Introduction

Per capita, the US spends more on health care than any other country worldwide,^1^ and hospitals receive the largest share of these dollars.^2^ In the US, hospitals are paid under the Inpatient Prospective Payment System, which was introduced in 1983 to reduce health care spending by predefining hospital reimbursement for given diagnoses and procedures.^3,4^ The Inpatient Prospective Payment System categorizes each discharged patient into a Diagnosis Related Group (DRG) with an assigned payment weight that reflects the average resources used to treat that condition.^5^ Payment weights for each DRG are updated annually to account for changes in operating and capital costs, labor and nonlabor inflation, hospital variability (eg, geography, presence or absence of quality programs, disproportionate share status), medical education, and potential upcoding.^6,7^

There are a total of 761 Medicare Severity–DRGs, which are organized into families (eg, heart failure) with 2 or 3 levels, most commonly with a base Medicare Severity–DRG (hereafter referred to as *DRG*) and 1 or 2 higher-complexity DRGs. Assignment to these latter DRGs occurs if 1 or more complications or comorbidities (CC) or major complications or comorbidities (MCC) are present. Of importance, hospital payment for DRGs with CCs or MCCs is often substantially greater. For example, payment for DRG 291 (heart failure and shock with MCC) is approximately twice that for DRG 293 (heart failure and shock without CC or MCC).

Because DRG assignment is based largely on diagnosis and procedure codes, accuracy of documentation is important. To that end, most US health care systems have implemented clinical documentation improvement programs to ensure that a patient’s clinical status is fully represented in the medical record and subsequently translated into appropriate coding categories.^8,9^ Clinical documentation improvement programs have focused much of their attention on more thorough capture of secondary diagnoses, which can inform the specific DRG assigned within a given DRG family, often with increased hospital payment.^10,11^

Assessment of the associations between DRGs, cost, and quality in the US goes back more than 3 decades.^12,13,14,15^ More recently, select DRGs have been used to evaluate key quality programs, such as the Hospital Value-Based Purchasing and Readmissions Reduction Programs.^16,17^ Less research as of late has been focused on associations between DRG shifts, case mix, and hospital payment.

We used all-payer data from the National Inpatient Sample (NIS) to examine the pattern of DRG assignment for the 20 highest-reimbursed DRG families. We aimed to (1) assess temporal trends in DRG assignment, particularly in terms of shifts to those with MCC within a given DRG family; (2) evaluate whether comorbidity scores and risk-adjusted mortality rates (RAMRs), as DRG-independent markers of disease severity, similarly changed; and (3) estimate changes in payment associated with these DRG shifts.

## Methods

### Population and Data Source

This retrospective cohort study used data from the Healthcare Cost and Utilization Project NIS, which is the largest publicly available, all-payer, inpatient hospital database in the US. Yearly data contain more than 7 million hospital stays, representing a stratified sample of approximately 20% of inpatient admissions to nonfederal hospitals (95% sampling frame of all hospital discharges in the US). The NIS does not contain unique patient identifiers; as such, each hospitalization is treated independently. This analysis included all inpatient admissions from January 1, 2012, to December 31, 2016, inclusive of the most current data available at the time of the analysis. This study was approved by the Providence St. Joseph Health institutional review board, with waiver of informed consent because data were deidentified. The study followed the Strengthening the Reporting of Observational Studies in Epidemiology (STROBE) reporting guideline.

Data on hospitalized patients younger than 18 years of age were excluded except those related to preterm birth (DRG 791-792: prematurity). Annual national prevalence was estimated using hospital weight and stratum information provided by the NIS. The annual estimated payment for each DRG was calculated using publicly available Centers for Medicare & Medicaid Services (CMS) weighted payments,^18^ multiplied by annual NIS weighted hospitalization counts. Estimated payments were summed for all DRGs within a given family, and the 20 DRG families with the highest payments in 2016 were selected for further analysis. Data were analyzed from July 10, 2018, to May 29, 2019.

### Study Outcomes

The primary outcome was the proportion of hospitalizations assigned to a DRG with MCC (or highest-complexity equivalent) in each DRG family. Secondary outcomes were comorbidity scores (using the Moore index^19^), RAMRs, and estimated payment.

The Moore index incorporates 29 comorbidity variables into a single index and represents a validated means to assess risk-adjusted mortality in large administrative data sets.^19^ To calculate the RAMR, multivariable logistic regression models stratified by DRG family were constructed with the following independent variables: age, sex, race, elective vs nonelective status, and Moore index score. The RAMR for each DRG was calculated using indirect standardization, defined as the ratio of the observed rate of the outcome to the expected rate of the outcome in the risk-adjusted model, multiplied by the unadjusted mortality rate observed in the whole study period.^20^ One of the DRG families (DRG 791-792: prematurity) did not have inpatient mortality data and was excluded from the RAMR calculation.

### Statistical Analysis

For each DRG family, the DRG coding percentage, Moore index score, and RAMR were summarized by quarter-year from the first quarter of 2012 to the fourth quarter of 2016. Quarter-year was chosen to account for seasonal variation in hospitalization, and the first quarter of 2012 was used as baseline. To compare temporal changes in these outcomes, we performed 3 linear regressions for each DRG, with change in DRG coding percentage, Moore index score, or RAMR as dependent variables and time as the independent variable. For graphical purposes, we fitted a LOESS model, a nonparametric model using locally weighted polynomial regression, to estimate the association between the aforementioned dependent variables and quarter-year across our study period. Results from the linear regression and LOESS models are presented as mean percentage change per quarter with 95% CIs.

To further assess the association between comorbidity burden and temporal changes in DRG assignment within a given DRG family, a multinomial logistic regression model was performed for the heart failure DRG family (DRGs 291, 292, and 293). The outcome was DRG assignment for each hospitalization, with the following independent variables: time (quarter-year), age, sex, race, elective vs nonelective status, and Moore index score. Results are presented as odds ratios (ORs) with 95% CIs.

To examine payment changes associated with DRG shifts over time, the average weighted payment for each DRG was multiplied by the coded percentage of that DRG in 2012 or 2016. All DRGs within a given family were summed and divided by 100 to calculate the average weighted payment per case in that year. By holding 2012 coding distribution constant and applying CMS weighted payments from 2016, we calculated the change in reimbursement. We then subtracted this from the 2016 average weighted payments to calculate the reimbursement difference owing to changes in DRG coding. Additional details are provided in eTable 1 and the eAppendix in the Supplement.

Furthermore, we conducted several sensitivity analyses. We examined whether our risk model choice (Moore index) was the primary driver of our findings by repeating the analyses with 3 different comorbidity models. The 29-variable Elixhauser model was originally developed to measure comorbidities in large administrative data sets.^21^ The Thompson index reduces the Elixhauser variables into a single score and has been validated in the NIS.^22^ The van Walravan index is a modified version of the Elixhauser model that also reduces the Elixhauser variables into a single score.^23^ Similar to our primary analysis, all models in the sensitivity analysis included the top 20 DRG families and the following covariates: age, sex, race, and elective vs nonelective status.

All hypothesis tests were 2-sided and *P* < .05 was considered statistically significant. The Tukey method was used for post hoc multiple comparison testing when needed. SAS, version 9.4 (SAS Institute Inc) and R, version 1.2.1335 (R Project for Statistical Computing) were used for all analyses.

## Results

### Sample Characteristics

Between 2012 and 2016, there were 62 167 976 hospitalizations for the 20 highest-reimbursed DRG families (Table 1). Patients included in this cohort were 32.9% male and 66.8% White, with a median age of 57 years (interquartile range, 31-73 years). These DRG families accounted for an estimated 12.9 million inpatient hospitalizations in 2016 (36.0% of all hospitalizations) and at least $115.4 billion in payment. Ten of the 20 DRG families were procedural; the remaining were medical. The top 2 DRG families, sepsis and lower extremity joint replacement, accounted for approximately one-quarter of the estimated payment. Volume and payment information (based on CMS weighting) for specific DRGs are provided in eTable 2 in the Supplement.

**Table 1.** Top 20 Reimbursed DRG Families in 2016^a^

Rank	DRG Family	Type	NIS unweighted case volume	NIS weighted case volume	Estimated payment, billions USD (% of top 20 total)
1	870-872: Sepsis	M	323 165	1 615 824	15.59 (13.5)
2	469-470: LE joint replacement	P	246 924	1 234 621	14.34 (12.4)
3	774-775: Vaginal delivery	M	485 836	2 429 178	8.11 (7.0)
4	3-4: ECMO or tracheostomy	P	19 143	95 715	7.86 (6.8)
5	853-855: Infectious diseases	P	56 197	280 985	6.77 (5.9)
6	765-766: Cesarean delivery	P	244 010	1 220 049	6.21 (5.4)
7	291-293: Heart failure	M	183 894	919 470	6.02 (5.2)
8	329-331: Bowel procedure	P	71 120	355 600	5.64 (4.9)
9	459-460: Spinal fusion	P	46 876	234 380	5.29 (4.6)
10	246-247: PCI with DES	P	71 732	358 660	4.73 (4.1)
11	193-195: Pneumonia	M	147 953	739 764	4.38 (3.8)
12	682-684: Renal failure	M	120 706	603 530	3.73 (3.2)
13	791-792: Prematurity	M	50 589	252 945	3.67 (3.2)
14	981-983: Extensive OR procedure	P	34 869	174 345	3.58 (3.1)
15	64-66: ICH or stroke	M	104 550	522 750	3.45 (3.0)
16	190-192: COPD	M	123 294	616 470	3.38 (2.9)
17	219-221: Valve surgery without cardiac catheterization	P	20 152	100 760	3.35 (2.9)
18	207-208: Respiratory disease	M	38 583	192 915	3.33 (2.9)
19	391-392: Esophageal and GI disorders	M	140 033	700 164	3.07 (2.7)
20	480-482: Hip and femur procedure except major joints	P	51 267	256 335	2.92 (2.5)
Total	NA	NA	2 580 893	12 904 460	115.39

Abbreviations: COPD, chronic obstructive pulmonary disease; DES, drug-eluting stent; DRG, Diagnosis Related Group; ECMO, extracorporeal membrane oxygenation; GI, gastrointestinal; ICH, intracranial hemorrhage; LE, lower extremity; M, medical; NA, not applicable; NIS, National Inpatient Sample; OR, operating room; P, procedural; PCI, percutaneous coronary intervention.

^a^ Based on Centers for Medicare & Medicaid Services weighted payments.

### Temporal Trends in DRGs With MCCs

**Figure 1.**
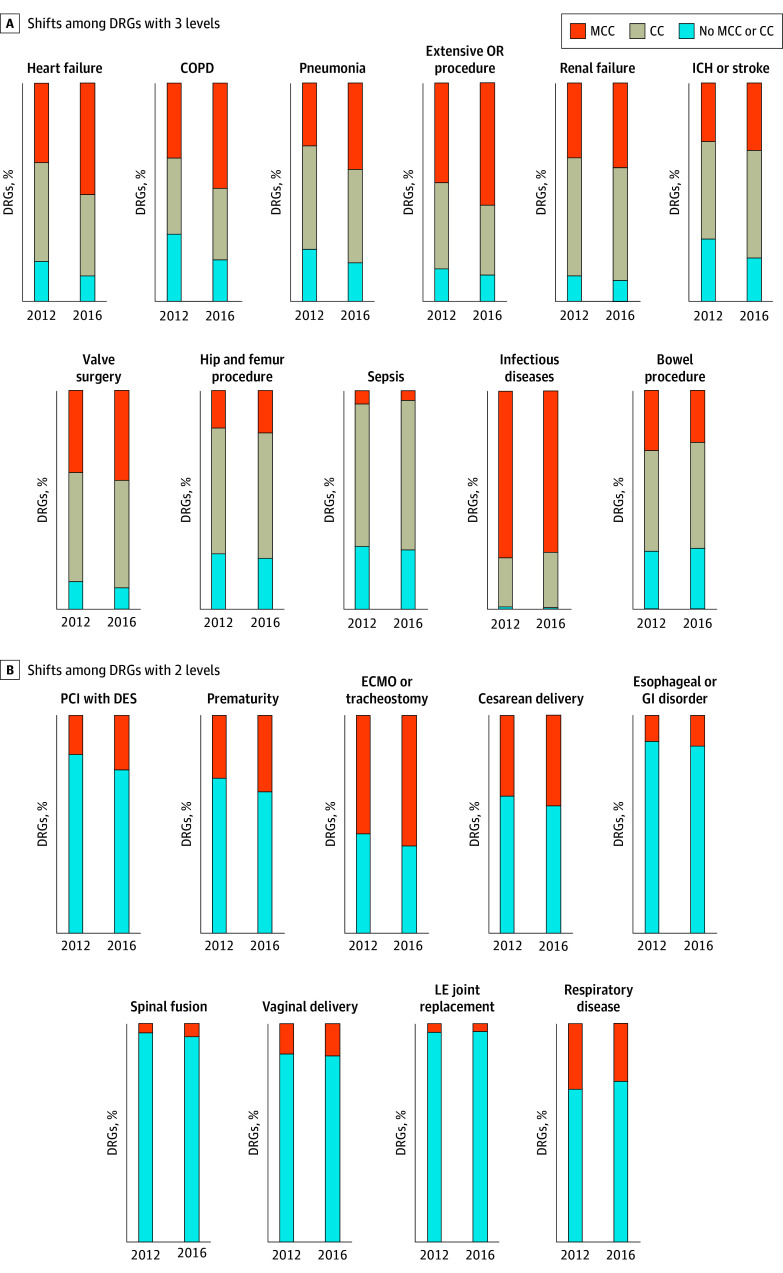
Changes in Diagnosis Related Groups (DRGs) Over Time DRG families are organized from those with the greatest positive change in DRG with major complication or comorbidity (MCC) (or highest-complexity equivalent) to the smallest change. CC indicates complication or comorbidity; COPD, chronic obstructive pulmonary disease; DES, drug-eluting stent; ECMO, extracorporeal membrane oxygenation; GI, gastrointestinal; ICH, intracranial hemorrhage; LE, lower extremity; OR, operating room; and PCI, percutaneous coronary intervention.

For 15 of the 20 DRG families (75%), the proportion of DRGs with MCC increased significantly over time (Figure 1). For example, for hip and femur procedures (a 3-level DRG family), 17.1% of admissions in 2012 and 19.4% in 2016 were assigned to a DRG with MCC. Shifts among the 3-level DRG families were most notable for heart failure, chronic obstructive pulmonary disease, and pneumonia. For percutaneous coronary intervention (a 2-level DRG family), 17.9% of admissions were assigned to a DRG with MCC in 2012 compared with 25.2% in 2016. Table 2 shows the mean percentage changes in DRGs with MCC in each family compared with the first quarter of 2012. Results for all DRGs are provided in eTable 3 in the Supplement.

**Table 2.** Changes in DRG Categories, Comorbidity Scores, and the Risk-Adjusted Mortality Rate for Admissions Assigned to a DRG With MCC

DRG	Change per quarter vs quarter 1 of 2012, % (95% CI)					*P* value
	Admissions assigned to a DRG with MCC	*P* value	Comorbidity score	*P* value	RAMR	
870: Sepsis with MV for >96 h	–1.91 (–2.48 to –1.34)	<.001	0.08 (–0.05 to 0.21)	.23	–0.10 (–0.37 to 0.17)	.47
469: LE joint replacement with MCC	–0.71 (–0.87 to –0.56)	<.001	–0.13 (–0.35 to 0.09)	.26	–1.19 (–1.9 to –0.49)	.004
774: Vaginal delivery with complicating diagnosis	0.45 (0.18 to 0.71)	.004	1.82 (1.34 to 2.31)	<.001	–1.04 (–3.47 to 1.39)	.41
3: ECMO or tracheostomy with MV for >96 h with major OR	0.7 (0.34 to 1.06)	.001	0.16 (–0.02 to 0.34)	.11	0.49 (0.03 to 0.95)	.051
853: Infectious diseases with MCC	–0.27 (–0.4 to –0.13)	.001	–0.17 (–0.31 to –0.04)	.02	–0.17 (–0.68 to 0.34)	.52
765: Cesarean delivery with CC or MCC	0.72 (0.62 to 0.83)	<.001	2.81 (2.47 to 3.16)	<.001	1.9 (–3.01 to 6.82)	.46
291: Heart failure with MCC	2.37 (1.64 to 3.09)	<.001	–0.07 (–0.21 to 0.07)	.35	–1.72 (–2.1 to –1.33)	<.001
329: Bowel procedure with MCC	–0.8 (–1.07 to –0.53)	<.001	0.09 (–0.07 to 0.25)	.29	–1.08 (–1.4 to –0.76)	<.001
459: Spinal fusion with MCC	2.3 (1.45 to 3.16)	<.001	–1.03 (–1.86 to –0.2)	.03	0.65 (–4.01 to 5.31)	.79
246: PCI with DES with MCC	2.58 (2.2 to 2.95)	<.001	1.02 (0.63 to 1.41)	<.001	1.76 (0.83 to 2.68)	.002
193: Pneumonia with MCC	2.29 (2.04 to 2.54)	<.001	–0.63 (–0.82 to –0.44)	<.001	–1.84 (–2.17 to –1.51)	<.001
682: Renal failure with MCC	0.74 (0.32 to 1.15)	.003	–0.23 (–0.41 to –0.06)	.02	–1.06 (–1.46 to –0.66)	<.001
791: Prematurity with major problems	1.29 (1.08 to 1.49)	<.001	–3.20 (–4.37 to –2.04)	<.001	NA^a^	NA^a^
981: Extensive OR procedure with MCC	1.18 (0.74 to 1.61)	<.001	–0.14 (–0.35 to 0.06)	.19	0.25 (–0.59 to 1.09)	.57
64: ICH or stroke with MCC	0.94 (0.66 to 1.21)	<.001	–0.23 (–0.4 to –0.07)	.01	–0.76 (–1.03 to –0.49)	<.001
190: COPD with MCC	2.27 (1.82 to 2.72)	<.001	0.07 (–0.13 to 0.28)	.49	–1.48 (–2.17 to –0.78)	<.001
219: Valve surgery without cardiac catheterization with MCC	0.6 (0.47 to 0.72)	<.001	0 (–0.19 to 0.19)	.99	–0.69 (–1.37 to –0.01)	.06
207: Respiratory disease with MV for >96 h	–0.84 (–1.18 to –0.49)	<.001	0.01 (–0.19 to 0.21)	.93	0.24 (–0.13 to 0.61)	.22
391: Esophageal and GI disorders with MCC	1.07 (0.73 to 1.41)	<.001	0.67 (0.55 to 0.79)	<.001	–1.17 (–2.07 to –0.27)	.02
480: Hip and femur procedure except major joint with MCC	0.76 (0.59 to 0.93)	<.001	–0.09 (–0.31 to 0.12)	.40	–0.58 (–1.21 to 0.06)	.09

Abbreviations: CC, complication or comorbidity; COPD, chronic obstructive pulmonary disease; DRG, Diagnosis Related Group; ECMO, extracorporeal membrane oxygenation; GI, gastrointestinal; ICH, intracranial hemorrhage; MCC, major complication or comorbidity; MV, mechanical ventilation; NA, not applicable; RAMR, risk-adjusted mortality rate.

^a^ There were no deaths in the prematurity DRG family; hospitalizations during which death occurred were coded into DRG-789: neonates, died or transferred to another acute care facility.

### Trends in DRG-Independent Measures of Disease Severity

Commensurate changes in disease severity were not observed over time. Moore comorbidity index scores decreased in 6 DRG families (30%), did not change in 10 (50%), and increased in 4 (20%) (Table 2). A similar pattern was seen with the RAMR, which significantly decreased in 8 of 19 DRG families (42%), did not change in 9 (47%), and increased in 2 (11%).

**Figure 2.**
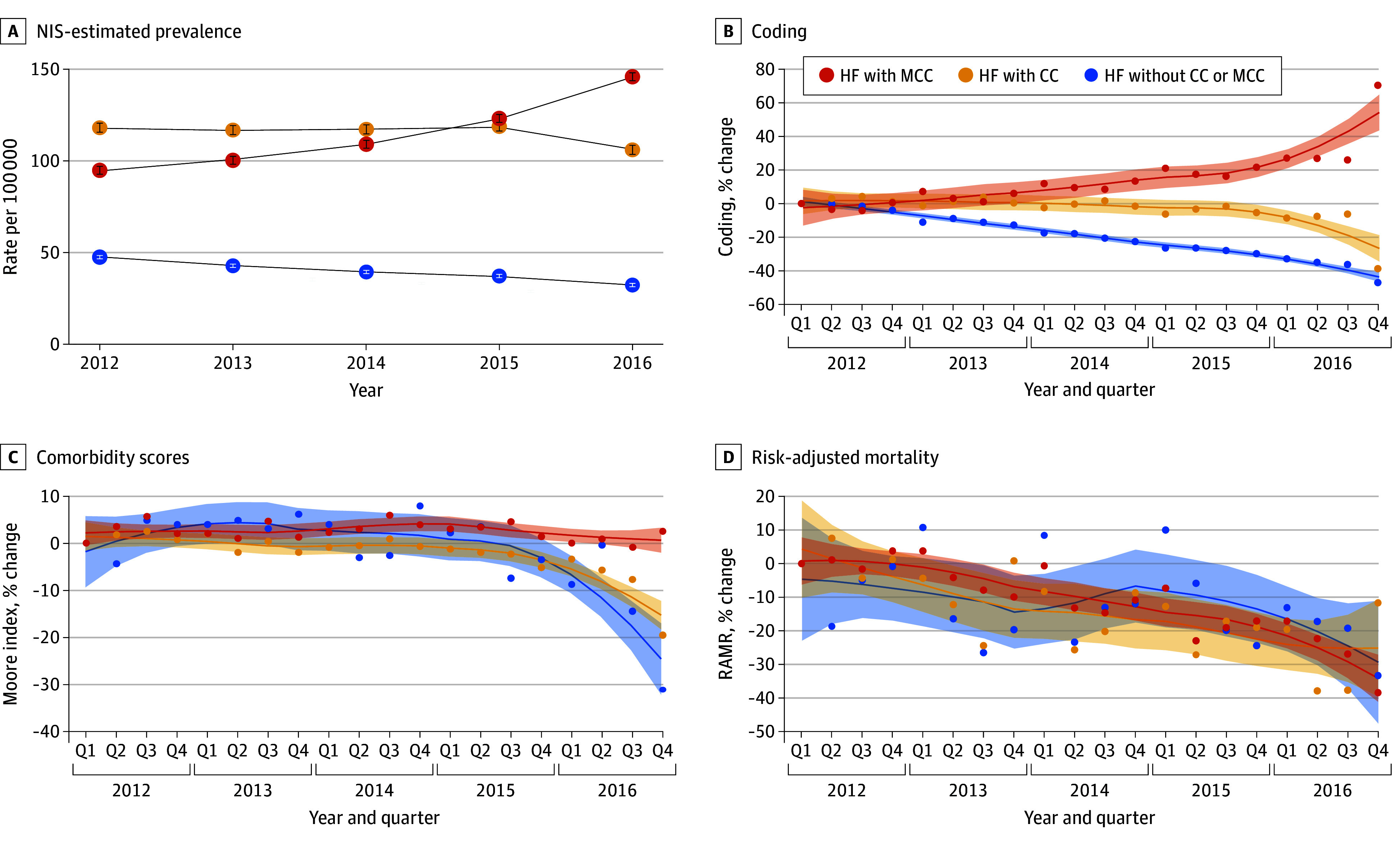
Temporal Trends in Heart Failure Prevalence, Coded Severity of Disease, Comorbidity Scores, and Risk-Adjusted Mortality Rate A, Error bars indicate 95% CIs. B-D, Shaded areas indicate 95% CIs for the comparison with quarter (Q) 1 of 2012. CC indicates complication or comorbidity; HF, heart failure; MCC, major complication or comorbidity; and RAMR, risk-adjusted mortality rate.

Figure 2 shows quarterly changes in the prevalence, assigned DRGs, comorbidity scores, and RAMRs for the heart failure DRG family. Hospitalizations for heart failure with higher Moore index comorbidity scores and those occurring later in the study period were also more likely to be coded as a DRG with MCC (eFigure 1 in the Supplement).

Consistent results were noted across risk models. Similar comorbidity trends were seen with the Moore-weighted, Thompson-weighted, and van Walraven–weighted index for those hospitalized with heart failure (eFigure 2 in the Supplement). Comparable trends for RAMR were seen with the Moore-weighted index and 29 Elixhauser comorbidities model (eFigure 3 in the Supplement), mimicking observed trends in the raw mortality rate.

### Associated Changes in Payment

From 2012 to 2016, the change in average weighted payment per case using data from CMS varied from a decrease of $873 for infectious diseases to an increase of $4897 for extracorporeal membrane oxygenation or tracheostomy with mechanical ventilation (Table 3). For 15 DRG families (75%), payment associated with shifts in DRG coding increased, ranging from $8 per case for vaginal delivery to $2057 per case for extracorporeal membrane oxygenation or tracheostomy. Overall, changes in assigned DRGs accounted for at least $1.2 billion more in payment in 2016 than would have been the case if the 2012 distribution of DRGs remained unchanged.

**Table 3.** Payment Changes Associated With Changes in Case Mix Over Time

DRG family	Average weighted payment per case based on case mix, USD			2012/2016 Payments, million USD per case/per family (% difference)^a^	
	2012	2016	Difference	Owing to CMS payment difference	Owing to coding difference
870-872: Sepsis	10 042	9649	–393	–49/–79 (–0.51)	–344/–556 (–3.57)
469-470: LE joint replacement	11 157	11 615	458	490/606 (4.22)	–33/–40 (–0.28)
774-775: Vaginal delivery	2906	3339	433	425/1.03 (12.72)^b^	8/20 (0.25)
3-4: ECMO or tracheostomy with MV for >96 h	76 947	81 844	4897	2839/272 (3.46)	2057/197 (2.51)
853-855: Infectious diseases	24 979	24 105	–873	–507/–143 (–2.11)	–366/–103 (–1.52)
765-766: Cesarean delivery	5155	5093	–62	–149/–181 (–2.92)	86/106 (1.7)
291-293: Heart failure	5897	6545	648	123/113 (1.88)	525/483 (8.03)
329-331: Bowel procedure	16 089	15 854	–236	348/124 (2.19)	–583/–207 (–3.68)
459-460: Spinal fusion	20 689	22 546	1857	1625/381 (7.21)	231/54 (1.03)
246-247: PCI with DES	11 439	13 187	1748	1302/467 (9.87)	446/160 (3.38)
193-195: Pneumonia	5583	6030	447	91/68 (1.54)	355/263 (6.01)
682-684: Renal failure	6188	6179	–9	–182/–110 (–2.95)	174/105 (2.81)
791-792: Prematurity	12 583	14 500	1917	1438/364 (9.92)	478/121 (3.3)
981-983: Extensive OR	19 389	20 755	1366	23/4 (0.11)	1343/234 (6.55)
64-66: ICH or stroke	6466	6593	127	–162/–85 (–2.46)	289/151 (4.38)
190-192: COPD	4978	5482	504	205/127 (3.75)	299/184 (5.45)
219-221: Valve surgery without cardiac catheterization	32 333	33 130	798	223/22 (0.67)	575/58 (1.73)
207-208: Respiratory disease	16 577	17 020	443	1043/201 (6.04)	–600/–116 (–3.48)
391-392: Esophageal and GI disorders	4062	4397	335	282/197 (6.42)	53/37 (1.21)
480-482: Hip and femur procedure except major joint	10 498	11 444	946	784/201 (6.89)	163/42 (1.43)
Total	NA	NA	NA	3.6 (3.10)^b^	1.2 (1.03)^b^

Abbreviations: CMS, Centers for Medicare & Medicaid Services; COPD, chronic obstructive pulmonary disease; DES, drug-eluting stent; DRG, Diagnosis Related Group; ECMO, extracorporeal membrane oxygenation; GI, gastrointestinal; ICH, intracranial hemorrhage; LE, lower extremity; NA, not applicable; OR, operating room; PCI, percutaneous coronary intervention.

^a^ Based on 2016 weighted case volume and 2016 payment.

^b^ Payments are in billions of USD.

## Discussion

In this cohort study, most of the 20 highest-reimbursed DRG families had a significant shift toward DRGs with MCC from 2012 to 2016. During the same time, there were inconsistent changes in comorbidity burden and a largely stable to improving RAMR. These shifts in coding were associated with at least $1.2 billion more in payment compared with what would have occurred without them.

Of importance, this study revealed an upward shift in admissions assigned to a DRG with MCC for most of the DRG families evaluated. Among 3-level DRG families, a shift to a DRG with MCC was most common, with a commensurate decrease in DRGs with no CC or MCC. Similar findings were observed among many of the 2-level DRG families. The reasons behind these findings are incompletely understood.

Shifts among the 3-level DRG families were most notable for heart failure, chronic obstructive pulmonary disease, and pneumonia, all of which are included in the Hospital Readmissions Reduction Program. This finding raises the possibility that some of the shifts may have been associated with greater focus on documentation in the context of financial incentives to improve performance for these particular conditions. There are other potential contributing factors, however. Concurrent with our observation of a shift to DRGs with MCC from 2012 to 2016, hospital adoption of electronic health record systems increased^24,25^ largely in association with meaningful-use incentives under the 2009 Health Information Technology for Economic and Clinical Health Act.^26^ Hospitals also made substantial investments in clinical documentation improvement programs^27^ cognizant of the reimbursement opportunities associated with a shift to more complex DRGs.^8^

Another finding of this study was the absence of a consistent increase in comorbidity burden and largely stable to decreasing RAMRs among admissions assigned to a DRG with MCC over time. Although the presence of only a single CC or MCC leads to a shift in the DRG assigned, the comorbidity assessments performed in our study may provide a more comprehensive measure of patient complexity. As such, our findings call into question whether the observed DRG shifts are a reflection of a more complex patient population or whether they instead reflect efforts by clinical documentation improvement programs to encourage documentation of secondary diagnoses (CCs and MCCs) that result in greater hospital reimbursement.

Similar observations have been noted in other studies examining health care quality,^28,29^ in which improved performance has at least in part been attributable to improved documentation. In a 1995 analysis of the New York Cardiac Surgery Reporting System, greater documentation of relevant comorbidities was considered to account for approximately 40% of observed improvement in the RAMR.^28^ More recently, a study using the Medicare Provider Analysis and Review file estimated that increases in coded severity of disease accounted for approximately 60% of improvement in readmission rates after implementation of the Hospital Readmissions Reduction Program.^29^ Analogous patterns were thought to underlie recent reports of decreasing risk-adjusted inpatient mortality rates for acute myocardial infarction, heart failure, and pneumonia within the Medicare population.^20^ Some of these changes were possibly associated with observed shifts in coding, with an increase in documented comorbidities or assignment of patients to a different DRG family altogether (eg, from a pneumonia DRG to a septicemia or severe sepsis DRG).^30^

In addition, DRG shifts were associated with a significant increase in estimated payment over time. Hospitals had an increase in reimbursement to care for patients who did not appear to be any sicker in terms of overall comorbidities or risk of death but were documented as such. The $1.2 billion in increased payment is likely an underestimate because our calculations used publicly available CMS payments applied to an all-payer population. Medicare was the primary payer for only 41.7% of admissions in this cohort. Although the NIS is an all-payer data set, payment rates for commercial payers were not available, and thus our estimation of payment was limited to that provided by the CMS. In prior analyses, payment to hospitals by commercial payers has largely exceeded that provided by the CMS for nearly all DRGs evaluated.^31^

In an era during which costs of care continue to increase and value-based care models are ubiquitous, there is a need to better understand the role that coding plays in increased health care spending. The CMS could consider moving from the current DRG system, which yields often significantly greater reimbursement by documenting a CC or MCC, to one in which patient risk is assessed more broadly. Support for this change would come from an increasing population of patients with more than 1 comorbidity and acknowledgment that the current DRG system does not adequately capture varying combinations of comorbidities.^32^ Most CMS quality and cost measures use a broader array of *International Classification of Diseases, Ninth Revision* and *International Statistical Classification of Diseases and Related Health Problems, Tenth Revision* diagnosis codes for both identification and risk adjustment. For example, the model used by CMS to risk adjust its readmission and mortality measures, along with its cost and utilization measures, contains 83 condition categories and basic demographic information.^33^

### Limitations

This study has limitations. First, because the NIS is an administrative data set, we could not assess appropriateness of the coded complexity of disease and thus DRG shifts. Significant geographic and DRG-specific variability in CC and MCC capture rates has been shown,^34^ with substantial ongoing opportunity to increase hospital reimbursement.^10,35^ The shift in DRGs observed in our study may only reflect a period of catch up to appropriately capture clinical risk. In the absence of audits or of confirmatory data from a second source, such as electronic health records, it is difficult to know what represents accurate diagnosis capture. Second, the NIS data set captures inpatient hospitalizations, not individual patients. Accordingly, we were unable to track other measures that may gauge disease severity (eg, readmission rates) or any postdischarge outcomes. Furthermore, because the NIS does not include other admission types (eg, outpatient, observation), additional shifts related to hospital status could not be assessed. Third, the estimated payments were based on NIS case volumes and CMS-weighted DRG payments and not on actual Inpatient Protective Payment System payment data. Fourth, our estimate of increased payment is only for the 20 highest-reimbursed DRG families (representing 36% of all inpatient admissions) and thus is likely to be an underestimate of total payment associated with DRG shifts. Fifth, our financial analysis applied Medicare payments to an all-payer population and likely provides an underestimate of hospital reimbursement from commercial payers.

## Conclusions

This cohort study revealed that, between 2012 and 2016, there were increases in the proportion of admissions assigned to a DRG with MCC in 15 of the 20 highest-reimbursed DRG families. This change was not associated with commensurate increases in disease severity as assessed by comorbidity burden or risk-adjusted mortality. These DRG shifts were associated with at least $1.2 billion in increased payment.

## Notes

Supplement. **eTable 1.** Full and abbreviated DRG titles **eAppendix.** Equations used for financial calculations in Table 3 **eTable 2.** Case volume and weighted payment for individual DRGs in the top 20 reimbursed DRG families in 2016 **eTable 3.** Changes in DRG categories, comorbidity scores, and risk-adjusted mortality for all DRGs in the top 20 reimbursed DRG families **eFigure 1.** Odds of categorization into the HF with MCC DRG for HF admissions **eFigure 2.** Comorbidity models sensitivity analysis **eFigure 3.** Risk-adjusted mortality rate sensitivity analysis
