# Supplementary material for: Trends in Diagnosis Related Groups for Inpatient Admissions and Associated Changes in Payment From 2012 to 2016
Source: JAMA Netw Open. 2020 Dec 7;3(12):e2028470. doi: 10.1001/jamanetworkopen.2020.28470 (PMC12527476; doi:10.1001/jamanetworkopen.2020.28470)
Supplement: Supplement. — eTable 1. Full and abbreviated DRG titles eAppendix. Equations used for financial calculations in Table 3 eTable 2. Case volume and weighted payment for individual DRGs in the top 20 reimbursed DRG families in 2016 eTable 3. Changes in DRG categories, comorbidity scores, and risk-adjusted mortality for all DRGs in the top 20 reimbursed DRG families eFigure 1. Odds of categorization into the HF with MCC DRG for HF admissions eFigure 2. Comorbidity models sensitivity analysis eFigure 3. Risk-adjusted mortality rate sensitivity analysis [file jamanetwopen-e2028470-s001.pdf]

Supplemental Online Content

Gluckman TJ, Spinelli KJ, Wang M, et al. Trends in Diagnosis Related Groups for inpatient admissions and associated changes in payment from 2012 to 2016. *JAMA Netw Open.* 2020;3(12):e2028470. doi:10.1001/jamanetworkopen.2020.28470

eTable 1. Full and abbreviated DRG titles
eAppendix. Equations used for financial calculations in Table 3

eTable 2. Case volume and weighted payment for individual DRGs in the top 20 reimbursed DRG families in 2016

eTable 3. Changes in DRG categories, comorbidity scores, and risk-adjusted mortality for all DRGs in the top 20 reimbursed DRG families

eFigure 1. Odds of categorization into the HF with MCC DRG for HF admissions

eFigure 2. Comorbidity models sensitivity analysis

eFigure 3. Risk-adjusted mortality rate sensitivity analysis

This supplemental material has been provided by the authors to give readers additional information about their work.

© 2020 Gluckman TJ et al. *JAMA Network Open.*

eTable 1: Full and abbreviated DRG titles

DRG	Full title	Abbreviated title
870	Septicemia or severe sepsis w MV >96 hours	Sepsis w MV >96 hrs
871	Septicemia or severe sepsis w/o MV >96 hours w MCC	Sepsis w/o MV >96 hrs w MCC
872	Septicemia or severe sepsis w/o MV >96 hours w/o MCC	Sepsis w/o MV >96 hrs w/o MCC
469	Major joint replacement or reattachment of lower extremity w MCC	LE joint replacement w MCC
470	Major joint replacement or reattachment of lower extremity w/o MCC	LE joint replacement w/o MCC
774	Vaginal delivery w complicating diagnoses	Vaginal delivery w complicating Dx
775	Vaginal delivery w/o complicating diagnoses	Vaginal delivery w/o complicating Dx
3	ECMO or trach w MV >96 hrs or PDX exc face, mouth & neck w major OR	ECMO or trach w MV >96 hrs w maj OR
4	Trach w MV >96 hrs or PDX exc face, mouth & neck w/o major OR	Trach w MV >96 hrs w/o maj OR
853	Infectious & parasitic diseases w OR procedure w MCC	Infectious diseases w MCC
854	Infectious & parasitic diseases w OR procedure w CC	Infectious diseases w CC
855	Infectious & parasitic diseases w OR procedure w/o CC/MCC	Infectious diseases w/o CC/MCC
765	Cesarean delivery w CC/MCC	Cesarean delivery w CC/MCC
766	Cesarean delivery w/o CC/MCC	Cesarean delivery w/o CC/MCC
291	Heart failure & shock w MCC	Heart failure w MCC
292	Heart failure & shock w CC	Heart failure w CC
293	Heart failure & shock w/o CC/MCC	Heart failure w/o CC/MCC
329	Major small & large bowel procedures w MCC	Bowel proc w MCC
330	Major small & large bowel procedures w CC	Bowel proc w CC
331	Major small & large bowel procedures w/o CC/MCC	Bowel proc w/o CC/MCC
459	Spinal fusion except cervical w MCC	Spinal fusion w MCC
460	Spinal fusion except cervical w/o MCC	Spinal fusion w/o MCC
246	Perc cardiovasc proc w drug-eluting stent w MCC or 4+ vessels/stents	PCI w DES w MCC
247	Perc cardiovasc proc w drug-eluting stent w/o MCC	PCI w DES w/o MCC
193	Simple pneumonia & pleurisy w MCC	Pneumonia w MCC
194	Simple pneumonia & pleurisy w CC	Pneumonia w CC
195	Simple pneumonia & pleurisy w/o CC/MCC	Pneumonia w/o CC/MCC
682	Renal failure w MCC	Renal failure w MCC
683	Renal failure w CC	Renal failure w CC
684	Renal failure w/o CC/MCC	Renal failure w/o CC/MCC
791	Prematurity w major problems	Prematurity w maj problems
792	Prematurity w/o major problems	Prematurity w/o maj problems
981	Extensive OR procedure unrelated to principal diagnosis w MCC	Extensive OR proc w MCC

© 2020 Gluckman TJ et al. *JAMA Network Open.*

982	Extensive OR procedure unrelated to principal diagnosis w CC	Extensive OR proc w CC
983	Extensive OR procedure unrelated to principal diagnosis w/o CC/MCC	Extensive OR proc w/o CC/MCC
64	Intracranial hemorrhage or cerebral infarction w MCC	ICH or stroke w MCC
65	Intracranial hemorrhage or cerebral infarction w CC or tPA in 24 hours	ICH or stroke w CC
66	Intracranial hemorrhage or cerebral infarction w/o CC/MCC	ICH or stroke w/o CC/MCC
190	Chronic obstructive pulmonary disease w MCC	COPD w MCC
191	Chronic obstructive pulmonary disease w CC	COPD w CC
192	Chronic obstructive pulmonary disease w/o CC/MCC	COPD w/o CC/MCC
219	Cardiac valve & other maj cardiothoracic proc w/o card cath w MCC	Valve surgery w/o card cath w MCC
220	Cardiac valve & other maj cardiothoracic proc w/o card cath w CC	Valve surgery w/o card cath w CC
221	Cardiac valve & other maj cardiothoracic proc w/o card cath w/o CC/MCC	Valve surgery w/o card cath w/o CC/MCC
207	Respiratory system diagnosis w ventilator support >96 hours	Respiratory disease with MV >96 hrs
208	Respiratory system diagnosis w ventilator support <96 hours	Respiratory disease with MV <96 hrs
391	Esophagitis, gastroent & misc digest disorders w MCC	Esoph and GI disorders w MCC
392	Esophagitis, gastroent & misc digest disorders w/o MCC	Esoph and GI disorders w/o MCC
480	Hip & femur procedures except major joint w MCC	Hip & femur proc except maj joint w MCC
481	Hip & femur procedures except major joint w CC	Hip & femur proc except maj joint w CC
482	Hip & femur procedures except major joint w/o CC/MCC	Hip & femur proc except maj joint w/o CC/MCC

CC = complication or comorbidity, COPD = chronic obstructive pulmonary disease, DES = drug-eluting stent, DRG = Diagnosis Related Group, Dx = diagnosis, ECMO = extracorporeal membrane oxygenation, GI = gastrointestinal, ICH = intracranial hemorrhage, LE = lower extremity, MCC = major complication or comorbidity, MV = mechanical ventilation, OR = operating room, PDX = principal diagnosis, PCI = percutaneous coronary intervention, tPA = tissue plasminogen activator

© 2020 Gluckman TJ et al. *JAMA Network Open.*

eAppendix: Equations used for financial calculations in Table 3

DRG family	Average weighted payment per case based on case mix			Payment Δ 2012-2016, per case / per family (percent Δ[a])	
	2012	2016	Δ 2012-2016	Due to CMS payment Δ	Due to coding Δ
	1	2	3	4/5(6)	7/8(9)

Equation 1: $= \sum_{i=1}^{n} \text{Yr 2012 coding\% of DRG}_i * \text{Yr 2012 weighted payment of DRG}_i / 100$ (n=2 or 3)

Equation 2: $= \sum_{i=1}^{n} \text{Yr 2016 coding\% of DRG}_i * \text{Yr 2016 weighted payment of DRG}_i / 100$ (n=2 or 3)

Equation 3: results of equation 2 **-** results of equation 1

Equation 4: $\sum_{i=1}^{n} \text{Yr 2012 coding\% of DRG}_i * \text{Yr 2016 weighted payment of DRG}_i$ (n=2 or 3) **-** results of equation 1

Equation 5: results of equation 4 *$\sum_{i=1}^{n} \text{Yr 2016 NIS national counts of DRG}_i$ (n=2 or 3)

Equation 6: $\frac{\text{results of equation 5}}{\sum_{i=1}^{n} \text{Yr 2016 NIS national counts of DRG}_i * \text{Yr 2016 weighted payment of DRG}_i \text{ (n=2 or 3)}} * 100$

Equation 7: results of equation 3 **-** results of equation 4

Equation 8: results of equation 7 *$\sum_{i=1}^{n} \text{Yr 2016 NIS national counts of DRG}_i$ (n=2 or 3)

Equation 9: $\frac{\text{results of equation 8}}{\sum_{i=1}^{n} \text{Yr 2016 NIS national counts of DRG}_i * \text{Yr 2016 weighted payment of DRG}_i \text{ (n=2 or 3)}} * 100$

© 2020 Gluckman TJ et al. *JAMA Network Open.*

eTable 2: Case volume and weighted payment for individual DRGs in the top 20 reimbursed DRG families in 2016

Rank	DRG	2016 NIS case volume	CMS weighted payment	Estimated total payment
1	870-Sepsis w MV >96 hrs	70,165	$ 32,131	2.25B
	871-Sepsis w/o MV >96 hrs w MCC	1,103,474	$ 9,799	10.81B
	872-Sepsis w/o MV >96 hrs w/o MCC	442,185	$ 5,699	2.52B
2	469-LE joint replacement w MCC	44,185	$ 18,017	0.80B
	470-LE joint replacement w/o MCC	1,190,436	$ 11,378	13.55B
3	774-Vaginal delivery w complicating Dx	359,820	$ 4,104	1.48B
	775-Vaginal delivery w/o complicating Dx	2,069,358	$ 3,206	6.63B
4	3-ECMO or trach w MV >96 hrs w maj OR	58,130	$ 96,514	5.61B
	4-Trach w MV >96 hrs w/o maj OR	37,585	$ 59,831	2.25B
5	853-Infectious diseases w MCC	207,705	$ 28,060	5.83B
	854-Infectious diseases w CC	71,020	$ 13,011	0.92B
	855-Infectious diseases w/o CC/MCC	2,260	$ 8,267	0.02B
6	765-Cesarean delivery w CC/MCC	506,750	$ 6,254	3.17B
	766-Cesarean delivery w/o CC/MCC	713,299	$ 4,267	3.04B
7	291-Heart failure w MCC	470,355	$ 8,095	3.81B
	292-Heart failure w CC	343,220	$ 5,306	1.82B
	293-Heart failure w/o CC/MCC	105,895	$ 3,683	0.39B
8	329-Bowel proc w MCC	84,665	$ 27,718	2.35B
	330-Bowel proc w CC	171,865	$ 13,945	2.40B
	331-Bowel proc w/o CC/MCC	99,070	$ 9,014	0.89B
9	459-Spinal fusion w MCC	14,140	$ 35,778	0.51B
	460-Spinal fusion w/o MCC	220,240	$ 21,710	4.78B
10	246-PCI w DES w MCC	90,350	$ 17,762	1.60B
	247-PCI w DES w/o MCC	268,310	$ 11,647	3.12B
11	193-Pneumonia w MCC	269,105	$ 7,795	2.10B

© 2020 Gluckman TJ et al. *JAMA Network Open.*

	194-Pneumonia w CC	317,859	$ 5,299	1.68B
	195-Pneumonia w/o CC/MCC	152,800	$ 3,887	0.59B
12	682-Renal failure w MCC	233,685	$ 8,246	1.93B
	683-Renal failure w CC	311,290	$ 5,141	1.60B
	684-Renal failure w/o CC/MCC	58,555	$ 3,428	0.20B
13	791-Prematurity w maj problems	88,825	$ 19,524	1.73B
	792-Prematurity w/o maj problems	164,120	$ 11,781	1.93B
14	981-Extensive OR proc w MCC	94,550	$ 26,528	2.51B
	982-Extensive OR proc w CC	55,855	$ 14,986	0.84B
	983-Extensive OR proc w/o CC/MCC	23,940	$ 9,629	0.23B
15	64-ICH or stroke w MCC	160,990	$ 9,471	1.52B
	65-ICH or stroke w CC	257,215	$ 5,790	1.49B
	66-ICH or stroke w/o CC/MCC	104,545	$ 4,140	0.43B
16	190-COPD w MCC	298,065	$ 6,329	1.89B
	191-COPD w CC	201,170	$ 5,095	1.02B
	192-COPD w/o CC/MCC	117,235	$ 3,997	0.47B
17	219-Valve surgery w/o card cath w MCC	42,000	$ 41,318	1.74B
	220-Valve surgery w/o card cath w CC	48,885	$ 27,918	1.36B
	221-Valve surgery w/o card cath w/o CC/MCC	9,875	$ 24,819	0.25B
18	207-Respiratory disease with MV >96 hrs	54,045	$ 29,242	1.58B
	208-Respiratory disease with MV <96 hrs	138,870	$ 12,602	1.75B
19	391-Esoph and GI disorders w MCC	96,360	$ 6,518	0.63B
	392-Esoph and GI disorders w/o MCC	603,804	$ 4,045	2.44B
20	480-Hip & femur proc except maj joint w MCC	48,410	$ 16,393	0.79B
	481-Hip & femur proc except maj joint w CC	142,485	$ 10,817	1.54B
	482-Hip & femur proc except maj joint w/o CC/MCC	65,440	$ 8,870	0.58B

Abbreviations as in eTable 1

© 2020 Gluckman TJ et al. *JAMA Network Open.*

eTable 3: Changes in DRG categories, comorbidity scores, and risk-adjusted mortality for all DRGs in the top 20 reimbursed DRG families

DRG	% change per quarter, relative to Q1-2012					
	% of admissions assigned to the DRG	P-value	Comorbidity score	P-value	RAMR	P-value
870- Sepsis w MV >96 hrs	-1.91 (-2.48,-1.34)	<0.0001	0.08 (-0.05, 0.21)	0.2292	-0.10 (-0.37, 0.17)	0.466
871-Sepsis w/o MV >96 hrs w MCC	0.23 (-0.03, 0.48)	0.0998	-0.32 (-0.40, -0.24)	<0.0001	-1.49 (-1.72, -1.26)	<0.0001
872-Sepsis w/o MV >96 hrs w/o MCC	-0.09 (-0.87, 0.69)	0.8193	-1.05 (-1.21, -0.89)	<0.0001	-2.64 (-3.35, -1.93)	<0.0001
469-LE joint replacement w MCC	-0.71 (-0.87, -0.56)	<.0001	-0.13 (-0.35, 0.09)	0.261	-1.19 (-1.9, -0.49)	0.0037
470-LE joint replacement w/o MCC	0.03 (0.02, 0.04)	<.0001	-33.75 (-40.09, -27.41)	<.0001	-1.52 (-2.68, -0.35)	0.02
774-Vaginal delivery w complicating Dx	0.45 (0.18, 0.71)	0.0038	1.82 (1.34, 2.31)	<.0001	-1.04 (-3.47, 1.39)	0.4108
775-Vaginal delivery w/o complicating Dx	-0.07 (-0.12, -0.03)	0.0038	2.02 (1.38, 2.65)	<.0001	N/A	N/A
3-ECMO or trach w MV >96 hrs w maj OR	0.7 (0.34, 1.06)	0.0012	0.16 (-0.02, 0.34)	0.1061	0.49 (0.03, 0.95)	0.0509
4-Trach w MV >96 hrs w/o maj OR	-0.8 (-1.21, -0.39)	0.0012	-0.04 (-0.25, 0.16)	0.6718	-0.74 (-1.25, -0.23)	0.0105
853-Infectious diseases w MCC	-0.27 (-0.4, -0.13)	0.0013	-0.17 (-0.31, -0.04)	0.017	-0.17 (-0.68, 0.34)	0.5171
854-Infectious diseases w CC	1.06 (-0.4, -0.13)	0.0005	-1.6 (-0.31, -0.04)	<.0001	-3.64 (-0.68, 0.34)	0.0072
855-Infectious diseases w/o CC/MCC	-1.6 (-2.73, -0.47)	0.0122	-4.28 (-9.93, 1.38)	0.1554	141.43 (-73.02, 355.89)	0.2125
765-Cesarean delivery w CC/MCC	0.72 (0.62, 0.83)	<.0001	2.81 (2.47, 3.16)	<.0001	1.9 (-3.01, 6.82)	0.4584
766-Cesarean delivery w/o CC/MCC	-0.42 (-0.49, -0.36)	<.0001	0.55 (0.01, 1.09)	0.0608	-2.05 (-4.81, 0.7)	0.1612
291-Heart failure w MCC	2.37 (1.64, 3.09)	<.0001	-0.07 (-0.21, 0.07)	0.3536	-1.72 (-2.1, -1.33)	<.0001
292-Heart failure w CC	-1.02 (-1.57, -0.48)	0.0016	-0.61 (-0.86, -0.35)	0.0002	-1.55 (-2.23, -0.87)	0.0003
293-Heart failure w/o CC/MCC	-2.24 (-2.41, -2.08)	<.0001	-0.91 (-1.47, -0.34)	0.0054	-0.88 (-1.77, 0.02)	0.0708
329-Bowel proc w MCC	-0.8 (-1.07, -0.53)	<.0001	0.09 (-0.07, 0.25)	0.292	-1.08 (-1.4, -0.76)	<.0001
330-Bowel proc w CC	0.29 (0.15, 0.43)	0.0007	-0.52 (-0.67, -0.36)	<.0001	-1.57 (-2.9, -0.24)	0.0327
331-Bowel proc w/o CC/MCC	0.32 (0.2, 0.45)	<.0001	-9.6 (-14.48, -4.72)	0.0012	-3.99 (-11.49, 3.52)	0.3114

© 2020 Gluckman TJ et al. *JAMA Network Open.*

459-Spinal fusion w MCC	2.3 (1.45, 3.16)	<.0001	-1.03 (-1.86, -0.2)	0.0259	0.65 (-4.01, 5.31)	0.7881
460-Spinal fusion w/o MCC	-0.1 (-0.14, -0.06)	<.0001	2.79 (0.96, 4.62)	0.0079	1.32 (-4.31, 6.94)	0.652
246-PCI w DES w MCC	2.58 (2.2, 2.95)	<.0001	1.02 (0.63, 1.41)	<.0001	1.76 (0.83, 2.68)	0.0015
247-PCI w DES w/o MCC	-0.56 (-0.64, -0.48)	<.0001	0.46 (-1.9, 2.82)	0.7094	1.51 (-0.04, 3.07)	0.0728
193-Pneumonia w MCC	2.29 (2.04, 2.54)	<.0001	-0.63 (-0.82, -0.44)	<.0001	-1.84 (-2.17, -1.51)	<.0001
194-Pneumonia w CC	-0.58 (-0.7, -0.47)	<.0001	-0.54 (-0.77, -0.32)	0.0001	-2.17 (-2.63, -1.7)	<.0001
195-Pneumonia w/o CC/MCC	-1.52 (-1.66, -1.38)	<.0001	-1.22 (-1.69, -0.74)	<.0001	-1.91 (-2.99, -0.82)	0.0028
682-Renal failure w MCC	0.74 (0.32, 1.15)	0.0027	-0.23 (-0.41, -0.06)	0.0174	-1.06 (-1.46, -0.66)	<.0001
683-Renal failure w CC	-0.27 (-0.46, -0.09)	0.009	-0.41 (-0.53, -0.28)	<.0001	-2.03 (-2.64, -1.42)	<.0001
684-Renal failure w/o CC/MCC	-1.04 (-1.56, -0.53)	0.0009	-0.72 (-0.94, -0.5)	<.0001	-1.72 (-3.18, -0.26)	0.033
791-Prematurity w maj problems	1.29 (1.08, 1.49)	<.0001	-3.20 (-4.37, -2.04)	<.0001	N/A[a]	N/A[a]
792-Prematurity w/o maj problems	-0.52 (-0.61, -0.44)	<.0001	0.47 (-0.76, 1.70)	0.4609	N/A[a]	N/A[a]
981-Extensive OR proc w MCC	1.18 (0.74, 1.61)	<.0001	-0.14 (-0.35, 0.06)	0.1946	0.25 (-0.59, 1.09)	0.5691
982-Extensive OR proc w CC	-1.07 (-1.51, -0.64)	0.0001	-1.1 (-1.43, -0.76)	<.0001	0.22 (-1.27, 1.7)	0.7778
983-Extensive OR proc w/o CC/MCC	-0.83 (-1.28, -0.37)	0.0022	-12.64 (-17.89, -7.38)	0.0002	2.57 (-3.09, 8.24)	0.3851
64-ICH or stroke w MCC	0.94 (0.66, 1.21)	<.0001	-0.23 (-0.4, -0.07)	0.0136	-0.76 (-1.03, -0.49)	<.0001
65-ICH or stroke w CC	0.57 (0.34, 0.81)	0.0001	-1.25 (-1.67, -0.82)	<.0001	-2.2 (-2.72, -1.69)	<.0001
66-ICH or stroke w/o CC/MCC	-1.78 (-2.09, -1.47)	<.0001	-6.26 (-8.18, -4.34)	<.0001	-1.91 (-2.55, -1.27)	<.0001
190-COPD w MCC	2.27 (1.82, 2.72)	<.0001	0.07 (-0.13, 0.28)	0.488	-1.48 (-2.17, -0.78)	0.0006
191-COPD w CC	-0.3 (-0.6, 0)	0.0698	-0.67 (-0.91, -0.43)	<.0001	-1.73 (-2.68, -0.79)	0.0021
192-COPD w/o CC/MCC	-2.24 (-2.45, -2.02)	<.0001	-2.86 (-4.26, -1.47)	0.0008	-1.6 (-2.55, -0.65)	0.004
219-Valve surgery w/o card cath w MCC	0.6 (0.47, 0.72)	<.0001	0 (-0.19, 0.19)	0.9943	-0.69 (-1.37, -0.01)	0.0607
220-Valve surgery w/o card cath w CC	-0.13 (-0.21, -0.04)	0.0088	0.41 (0.2, 0.62)	0.0012	-1.0 (-2.51, 0.5)	0.2078
221-Valve surgery w/o card cath w/o CC/MCC	-1.28 (-1.53, -1.04)	<.0001	2.42 (0.19, 4.65)	0.0474	-0.49 (-4, 3.03)	0.7895
207-Respiratory disease with MV >96 hrs	-0.84 (-1.18, -0.49)	0.0001	0.01 (-0.19, 0.21)	0.9307	0.24 (-0.13, 0.61)	0.222

© 2020 Gluckman TJ et al. *JAMA Network Open.*

208-Respiratory disease with MV <96 hrs	0.38 (0.23, 0.54)	0.0001	-0.01 (-0.19, 0.17)	0.8928	-0.27 (-0.57, 0.03)	0.0956
391-Esoph and GI disorders w MCC	1.07 (0.73, 1.41)	<.0001	0.67 (0.55, 0.79)	<.0001	-1.17 (-2.07, -0.27)	0.0203
392-Esoph and GI disorders w/o MCC	-0.15 (-0.2, -0.1)	<.0001	0.11 (-0.1, 0.31)	0.3282	2.63 (0.47, 4.79)	0.0279
480-Hip & femur proc except maj joint w MCC	0.76 (0.59, 0.93)	<.0001	-0.09 (-0.31, 0.12)	0.3996	-0.58 (-1.21, 0.06)	0.0915
481-Hip & femur proc except maj joint w CC	-0.01 (-0.07, 0.05)	0.7517	-0.18 (-0.34, -0.02)	0.0381	-0.4 (-1.68, 0.89)	0.5523
482-Hip & femur proc except maj joint w/o CC/MCC	-0.51 (-0.62, -0.41)	<.0001	-0.97 (-1.58, -0.36)	0.0061	-0.65 (-2.97, 1.66)	0.5862

a. There were no deaths in the prematurity DRG family; hospitalizations that resulted in death were coded into DRG-789-Neonates, died or transferred to another acute care facility.

Data presented as % change (95% CI)

RAMR = risk-adjusted mortality rate, additional abbreviations as in eTable 1

© 2020 Gluckman TJ et al. *JAMA Network Open.*

eFigure 1: Odds of categorization into the HF with MCC DRG for HF admissions

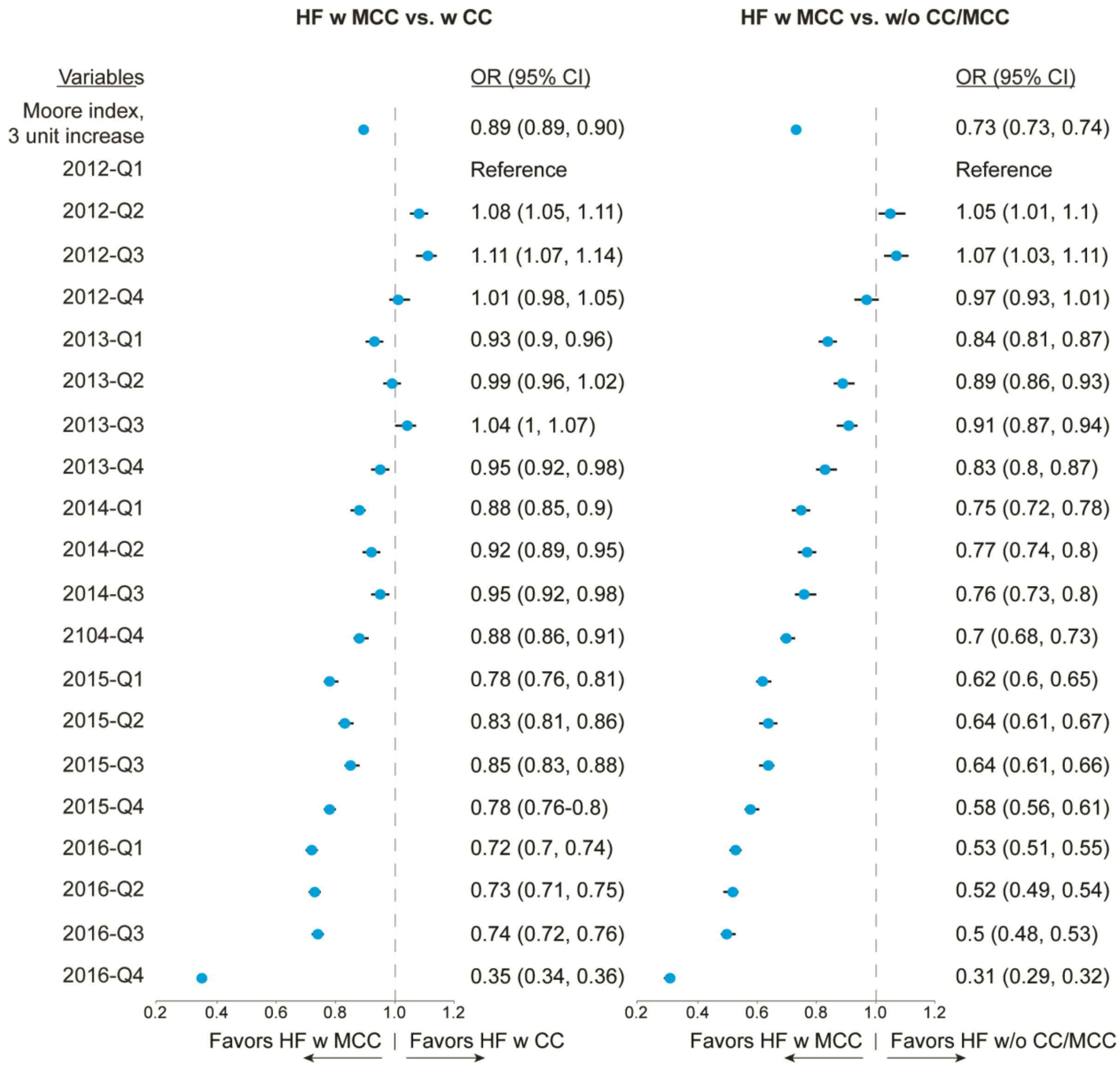

For all NIS HF admissions in the study period, odds ratios (OR) and 95% confidence intervals (CI) for categorization as HF with MCC vs. HF with CC (left column) or HF with MCC vs. HF w/o CC/MCC (right column). Independent variables included in the model were time (quarter-year), Moore index, age, sex, race, and elective vs non-elective status. CI = confidence interval, HF = heart failure, OR = odds ratio, Q = quarter, additional abbreviations as in eTable 1.

© 2020 Gluckman TJ et al. *JAMA Network Open.*

eFigure 2: Comorbidity models sensitivity analysis

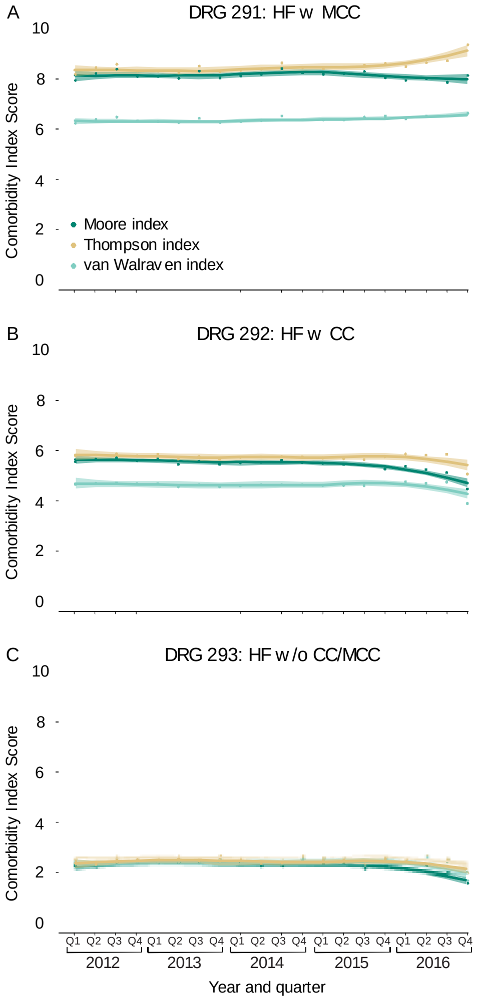

Comparison of trends over time for the Moore, Thompson, and van Walraven indices for (A) DRG 291, (B) DRG 292, and (C) DRG 293. Abbreviations as in eTable 1 and eFigure 1.

eFigure 3: Risk-adjusted mortality rate sensitivity analysis

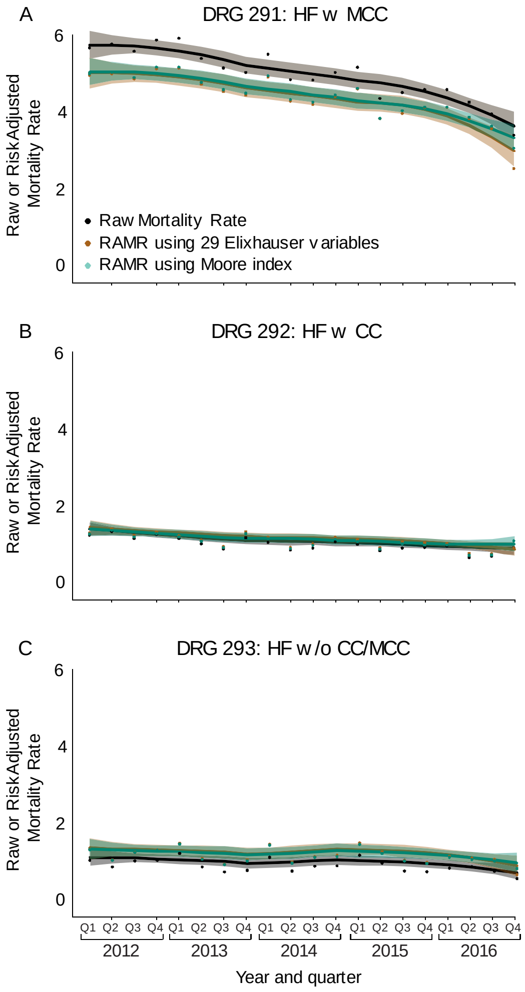

Comparison of trends over time for the raw mortality rate, risk-adjusted mortality rate using the 29 Elixhauser variables, and risk-adjusted mortality rate using the Moore index for (A) DRG 291, (B) DRG 292, and (C) DRG 293. Abbreviations as in eTable 1 and eTable 3.

© 2020 Gluckman TJ et al. *JAMA Network Open.*
